# The use of resazurin as a novel antimicrobial agent against *Francisella tularensis*

**DOI:** 10.3389/fcimb.2013.00093

**Published:** 2013-12-06

**Authors:** Deanna M. Schmitt, Dawn M. O'Dee, Brianna N. Cowan, James W.-M. Birch, Leanne K. Mazzella, Gerard J. Nau, Joseph Horzempa

**Affiliations:** ^1^Department of Natural Sciences and Mathematics, West Liberty UniversityWest Liberty, WV, USA; ^2^Department of Microbiology and Molecular Genetics, University of Pittsburgh School of MedicinePittsburgh, PA, USA; ^3^Department of Medicine – Division of Infectious Diseases, University of Pittsburgh School of MedicinePittsburgh, PA, USA; ^4^Center for Vaccine Research, University of Pittsburgh School of MedicinePittsburgh, PA, USA

**Keywords:** *Francisella*, resazurin, antibiotic, *Neisseria*, resorufin, tularemia, antibacterial, macrophages

## Abstract

The highly infectious and deadly pathogen, *Francisella tularensis*, is classified by the CDC as a Category A bioterrorism agent. Inhalation of a single bacterium results in an acute pneumonia with a 30–60% mortality rate without treatment. Due to the prevalence of antibiotic resistance, there is a strong need for new types of antibacterial drugs. Resazurin is commonly used to measure bacterial and eukaryotic cell viability through its reduction to the fluorescent product resorufin. When tested on various bacterial taxa at the recommended concentration of 44 μM, a potent bactericidal effect was observed against various *Francisella* and *Neisseria* species, including the human pathogens type A *F. tularensis* (Schu S4) and *N. gonorrhoeae*. As low as 4.4 μM resazurin was sufficient for a 10-fold reduction in *F. tularensis* growth. In broth culture, resazurin was reduced to resorufin by *F. tularensis*. Resorufin also suppressed the growth of *F. tularensis* suggesting that this compound is the biologically active form responsible for decreasing the viability of *F. tularensis* LVS bacteria. Replication of *F. tularensis* in primary human macrophages and non-phagocytic cells was abolished following treatment with 44 μM resazurin indicating this compound could be an effective therapy for tularemia *in vivo*.

## Introduction

*Francisella tularensis* is the causative agent of the zoonotic disease tularemia (Oyston et al., 2004). This disease is endemic in North America, Europe, and Asia with outbreaks often associated with the handling of infected animals or transmission by arthropod vectors (Sjostedt, 2007; Oyston, 2008). The Centers for Disease Control and Prevention has categorized *F. tularensis* as a Category A bioterrorism agent due to its ease of aerosolization, low infectious dose, and high mortality rate (McLendon et al., 2006). Inhalation of fewer than 10 bacteria results in an acute pneumonia that is lethal in 30–60% of individuals if left untreated (Dennis et al., 2001; McLendon et al., 2006).

When implemented early in infection, antibiotics are effective at reducing the case fatality rate for tularemia (Dennis et al., 2001; Barry et al., 2009). Aminoglycosides are commonly prescribed, specifically streptomycin or gentamicin, although tetracyclines and fluoroquinolones also have antimicrobial activity against *F. tularensis* (Nigrovic and Wingerter, 2008; Oyston, 2009). Tetracyclines, however, are associated with high relapse rates in tularemia patients (Thomas and Schaffner, 2010). Since this disease is often misdiagnosed due to its generic symptoms, antibiotic treatment may be delayed resulting in reduced survival (Barry et al., 2009). There is also a potential for the introduction of antibiotic-resistant strains (Oyston, 2009). While a tularemia vaccine is available (live vaccine strain, LVS), it is not currently licensed for use in the United States (Conlan and Oyston, 2007). Due to these concerns, there is an increased interest in developing alternative therapies for tularemia.

**Figure 1:**
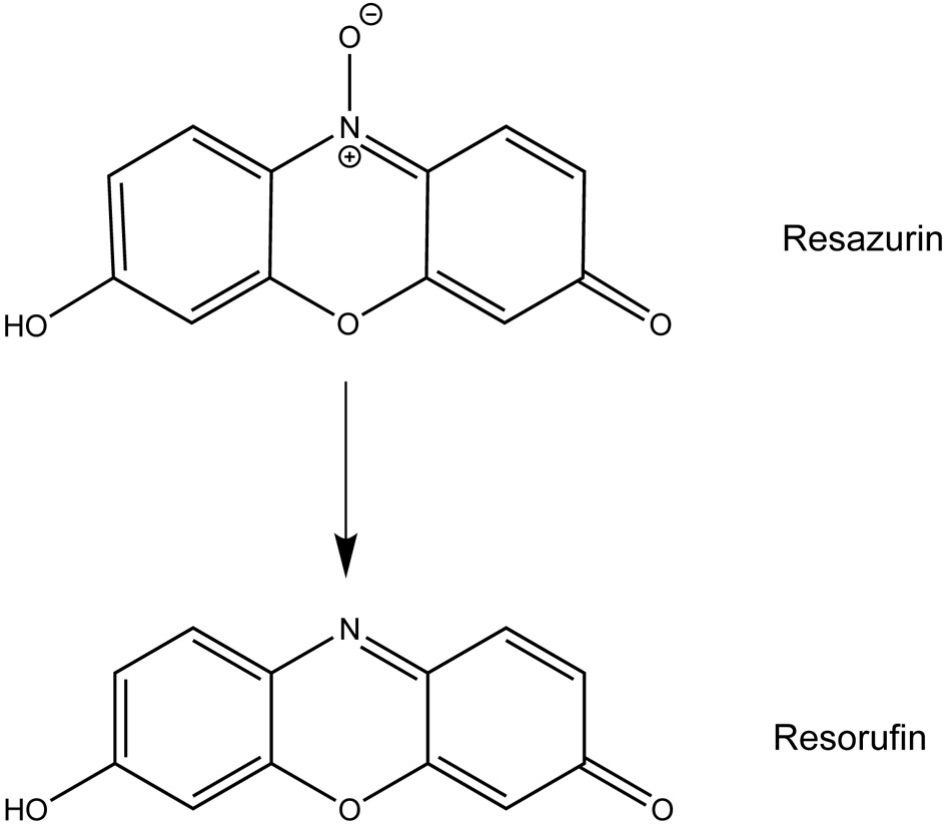
**The reduction of resazurin to resorufin**.

Resazurin, the active compound in alamarBlue^®^, has been used for decades to measure proliferation and cytotoxicity in prokaryotic and eukaryotic cells (Page et al., 1993; Ahmed et al., 1994; O'Brien et al., 2000). In metabolically active cells, this blue, non-fluorescent dye is reduced to the pink and highly fluorescent compound resorufin allowing for a quantitative measurement of cell viability (Figure 1) (O'Brien et al., 2000). Upon use of resazurin to monitor *F. tularensis* viability in culture at the recommended concentration of 44 μM, we discovered a novel antibacterial activity for this compound. Resazurin, and its reduced derivative resorufin, decreased the number of viable *F. tularensis* bacteria in broth culture by 100-fold after 1 day of cultivation. Growth of other bacterial genera was unaffected by this compound with the exception of *Neisseria* species, particularly the human pathogen *N. gonorrhoaea*. Resazurin also limited replication of *F. tularensis* in human macrophages and non-phagocytic cells highlighting the potential use of this compound as a novel antibacterial therapy *in vivo*.

## Materials and methods

### Bacterial strains and growth conditions

Bacterial strains used in this study are listed in Table 1. Bacteria grown on solid media were used to inoculate chocolate II agar plates or TSBc [trypticase soy broth (BD Biosciences) containing 0.1% L-cysteine hydrochloride monohydrate (Fisher)] supplemented with or without various concentrations of resazurin sodium salt (Acros Organics, dissolved in water) or resorufin (Tokyo Chemical Industries, dissolved in dimethyl sulfoxide). Chocolate II agar plates were incubated at 37°C with 5% CO_2_ (*Francisella* species, *Neisseria* species, and *Listeria monocytogenes*) or without CO_2_ (all other bacteria) for 1–3 days while broth cultures were incubated at 37°C with shaking. All work with Schu S4 was conducted under BSL-3 conditions at the University of Pittsburgh with approval from the CDC Select Agent Program.

**Table 1:** **Bacterial strains used in this study**.

**Bacterial strain**	**Source**
*Francisella tularensis* subsp. *holarctica* live vaccine strain	Karen Elkins
*F. tularensis* subsp. *tularensis* Schu S4 (NR-643)	NIH BEI Resources Repository^a^
*F. novicida* U112	Karen Elkins
*F. philomiragia* (ATCC 25018)	ATCC
*Neisseria gonorrhoeae* (ATCC 9793)	ATCC
*N. polysaccharea* (ATCC 43768)	ATCC
*N. sicca* (ATCC 9913)	ATCC
*Acinetobacter baumannii* (ATCC 19606)	ATCC
*Pseudomonas aeruginosa* 1244	Peter Castric
*Escherichia coli*	WLU-MCC^b^
*Salmonella typhimurium*	WLU-MCC^b^
*Staphylococcus aureus*	WLU-MCC^b^
*Listeria monocytogenes* EGD	Douglas Drevets
*Klebsiella pneumoniae*	WLU-MCC^b^
*Streptococcus pneumoniae*, clinical isolate	Robert Shanks

a National Institutes of Health Biodefense and Emerging Infections (NIH BEI) Research Resources Repository, National Institute of Allergy and Infectious Diseases.

b WLU-MCC = West Liberty University Microbiology Culture Collection, bacterial species routinely verified by standard metabolic and physiological tests.

### Reduction of resazurin to resorufin by *F. tularensis*

*F. tularensis* was cultured in TSBc supplemented with 44 μM resazurin at 37°C with shaking for 24 h. At select timepoints, a Spectronic 200 Spectrophotometer was used to measure the absorbance at 600 nm and 570 nm to detect the presence of resazurin and resorufin, respectively. The ratio of these two optical densities was used to evaluate reduction of resazurin to resorufin over time.

### Growth of *F. tularensis* in human macrophages and HEK293 cells

Human monocytes purified from buffy coats from blood donations (New York Blood Center, Long Island City, NY and the Central Blood Bank, Pittsburgh, PA) were differentiated into macrophages as described previously (Carlson et al., 2007, 2009; Horzempa et al., 2008a,b, 2010; Robinson and Nau, 2008; Robinson et al., 2010, 2012; Russo et al., 2011; Schmitt et al., 2012). Macrophages were then washed and resuspended in Dulbecco's modified Eagle's medium (DMEM) supplemented with 1% human serum AB (Gemini Bio-Products), 25 mM HEPES (Cellgro), and 1% glutamine dipeptide (Fisher Scientific). HEK293 cells (ATCC CRL-1573), a non-phagocytic kidney epithelial cell line (Tachado et al., 2007), were cultured in DMEM supplemented with 10% fetal bovine serum (Gibco), 25 mM HEPES, and 1% glutamine dipeptide with 100 U/ml penicillin-streptomycin (Cellgro). HEK293 cells were passaged at least once without antibiotics prior to use. To assess intracellular growth, gentamicin protection assays were performed (Small et al., 1987). Macrophages and HEK293 cells were seeded in Primaria 96-well culture dishes (BD Biosciences) at a density of 5 × 10^4^ cells/well. *F. tularensis* bacteria recovered from broth cultures described above were adjusted to an OD_600_ of 0.3 (approximately 1.5 × 10^9^ CFU/ml) and diluted to achieve a multiplicity of infection (MOI) of 500. The actual MOI was measured by plating serial dilutions of the inoculum on chocolate II agar plates. Cells were incubated with this MOI for 2 h yielding an infection rate of >80% (Carlson et al., 2007; Horzempa et al., 2008a) either in the absence or presence of 44 μM resazurin. After this time period, cells were incubated with gentamicin (100 μg/ml) for 30 min to kill extracellular bacteria and then washed twice with warm Hanks balanced salt solution (Cellgro). Fresh culture media with or without resazurin (44 μM) was then added and cells were incubated for another 22 h at 37°C with 5% CO_2_. At the indicated timepoints, cells were lysed with 0.02% sodium dodecyl sulfate and viable CFU were measured as described below.

### Enumeration of bacteria

At the indicated timepoints, a portion of the *F. tularensis* broth cultures or human cell lysates were serially diluted and plated onto chocolate II agar plates. Plates were incubated at 37°C at 5% CO_2_ for 2–3 days and individual colonies were enumerated. The limit of detection was 100 CFU per ml for broth culture or per 5 × 10^4^ cells for intracellular growth assays.

### Analytical methods

Statistically significant differences in bacterial number were determined by a Student's *t*-test or ANOVA followed by a Dunnett's or Bonferroni *post-hoc* test (GraphPad Prism 5). The chemcial structures of resazurin, resorufin, and acridine were drawn using ChemDraw Pro 13.0 for comparative analysis.

## Results

### Resazurin selectively inhibits growth of francisella and neisseria species

**Figure 2:**
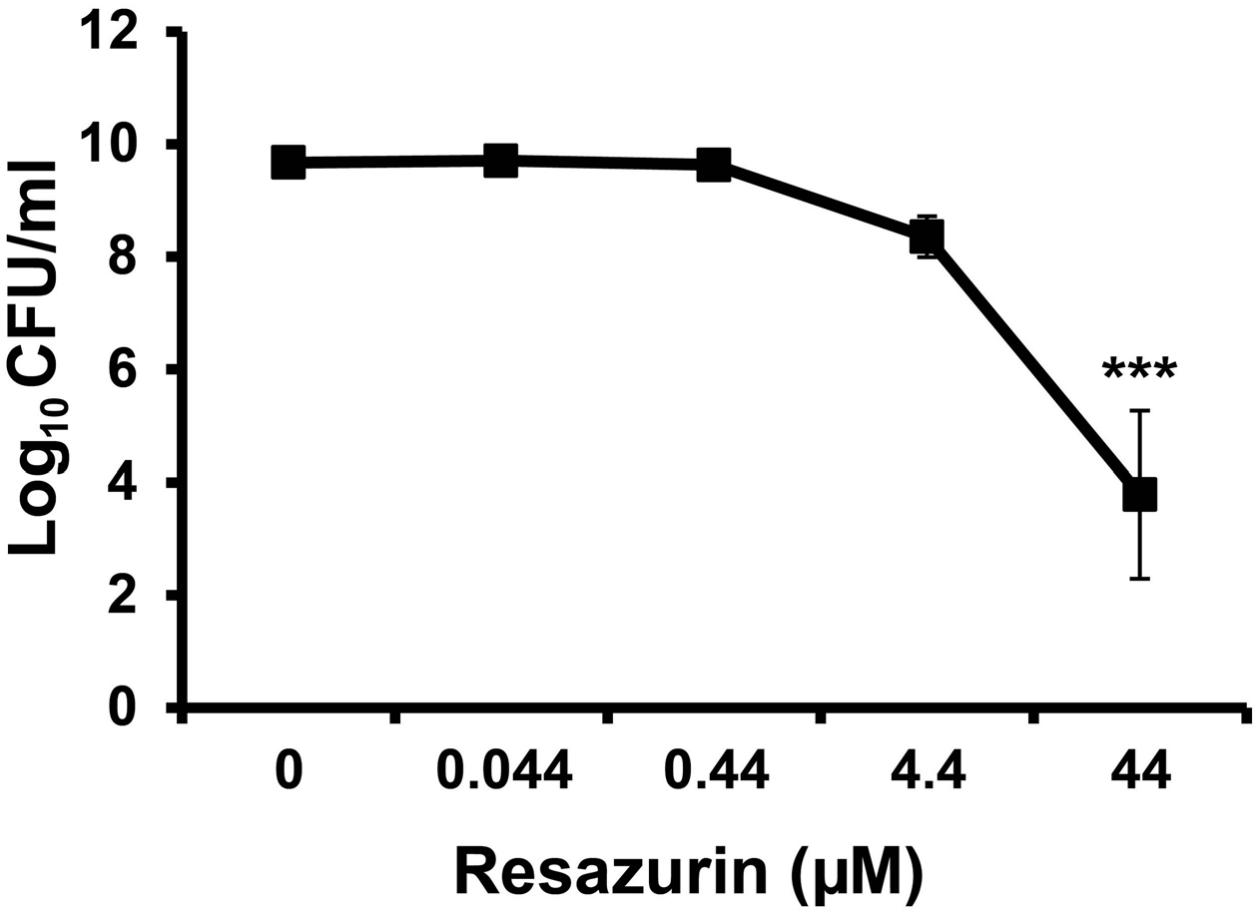
**Resazurin has an antimicrobial effect on *F. tularensis* LVS**. Bacteria were cultivated in tryptic soy broth supplemented with 0.1% cysteine HCl (TSBc) in the presence or absence of resazurin at the designated concentrations for 24 h. Cultures were then diluted and plated to determine the number of viable *F. tularensis* LVS bacteria 24 h post inoculation. Data shown are mean ± s.e.m. from three individual experiments. The limit of detection was 100 CFU per ml. Statistically significant differences in growth post-inoculation were determined by One-Way ANOVA followed by Dunnett's *post-hoc* test (^***^p < 0.001 compared to 0 μM resazurin).

Resazurin has been used previously as an indicator of cell growth for various bacterial species (Mendoza-Aguilar et al., 2012; Bassett et al., 2013; Bauer et al., 2013; Lall et al., 2013). We were interested in using this compound to monitor viability of *F. tularensis* in broth culture over time. Unexpectedly, no viable bacteria were detected 24 h post-inoculation following inclusion of resazurin in TSBc cultures of *F. tularensis* LVS at the concentration recommended by the manufacturer (44 μM) (data not shown). This concentration of resazurin had no effect on the growth of *E. coli* or *P. aeruginosa* cultivated in the same medium (data not shown). The antimicrobial activity of resazurin on *F. tularensis* LVS was not specific to TSBc as these bacteria were also unable to grow on chocolate II agar plates containing this compound (Table 2) as well as a chemically defined medium (data not shown). Lowering the resazurin concentration to as little as 4.4 μM still resulted in a 10-fold reduction in viable *F. tularensis* LVS compared to growth medium alone (Figure 2). These data suggest that resazurin exhibits bactericidal activity against *F. tularensis*.

**Table 2:** **Resazurin inhibits the growth of *Francisella* and *Neisseria* species on chocolate II agar**.

	**Resazurin Concentration**	
**Bacterial strain**	**0 μM**	**44 μM**
*Francisella tularensis* Schu S4	+	−
*F. tularensis* LVS	+	−
*F. novicida*	+	+
*F. philomiragia*	+	+
*Neisseria gonorrhoeae*	+	−
*N. polysaccharea*	+	−
*N. sicca*	+	−
*Acinetobacter baumannii*	+	+
*Pseudomonas aeruginosa* 1244	+	+
*Escherichia coli*	+	+
*Salmonella typhimurium*	+	+
*Staphylococcus aureus*	+	+
*Listeria monocytogenes*	+	+
*Klebsiella pneumoniae*	+	+
*Streptococcus pneumoniae*	+	+

+, robust growth.

−, no growth.

To determine whether the antibacterial effect of resazurin was specific to this organism, an assortment of bacteria from diverse taxa were plated on chocolate II agar plates supplemented with resazurin. All bacterial species tested were able to grow in the presence of 44 μM resazurin except *F. tularensis* and *Neisseria* species (Table 2). These data suggest that resazurin is an antimicrobial compound with specificity for *F. tularensis* and *Neisseria* species bacteria.

### Reduction of resazurin to resorufin does not alter its antibacterial activity

**Figure 3:**
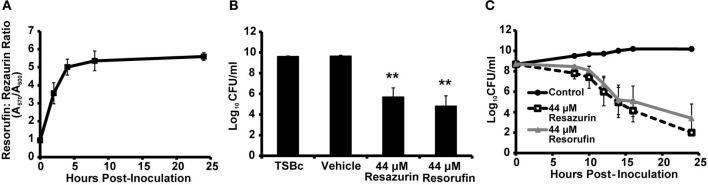
***F. tularensis* LVS reduces resazurin to resorufin which also inhibits bacterial growth**. *F. tularensis* LVS bacteria were cultivated in TSBc that was treated with resazurin, resorufin, or the solvent vehicle (DMSO). **(A)** Conversion of resazurin (absorbance at 600 nm) to resorufin (absorbance at 570 nm) in *F. tularensis* LVS cultures over 24 h. **(B,C)** *F. tularensis* LVS cultures were diluted and plated to determine the number of viable bacteria at 24 h **(B)** or the indicated timepoints **(C)**. Data shown are mean ± s.e.m. from three individual experiments. The limit of detection was 100 CFU per ml. Statistically significant differences in growth post-inoculation were determined by **(B)** One-Way ANOVA followed by Dunnett's *post-hoc* test (^**^p < 0.01 compared to TSBc) or **(C)** Two-Way ANOVA followed by Bonferroni comparison of means (*p* < 0.05 for Control vs. Resazurin and Control vs. Resorufin for 12–24 h).

As previously mentioned, viable cells are capable of converting resazurin to resorufin. To determine whether this reduction was occurring in *F. tularensis* LVS cultures, the ratio of resorufin to resazurin was measured over time using the optical densities at 570 nm (resorufin) and 600 nm (resazurin). The ratio of resorufin to resazurin increased 3-fold within 2 h of inoculation with *F. tularensis* LVS, reaching a maximum ratio of 5 four hours post-inoculation which was maintained for the remainder of the 24 h period (Figure 3A). This suggested *F. tularensis* LVS was reducing resazurin to resorufin. Therefore, we determined if resorufin also exhibited antibacterial activity against *F. tularensis*. Following 24 h of culture in the presence of resorufin, the number of *F. tularensis* LVS bacteria was significantly reduced compared to growth medium supplemented with vehicle alone (Figure 3B). A similar decrease in bacterial number was observed following incubation of *F. tularensis* LVS with resazurin (Figure 3B). These data suggest resorufin is also bactericidal against *F. tularensis*. To evaluate whether resazurin must first be converted to resorufin to exhibit antibacterial activity, we measured viable *F. tularensis* LVS bacteria over time grown in the presence of resazurin and resorufin. In cultures treated with resazurin, reduction of this compound to resorufin was observed as early as 2 h post-inoculation (Figure 3A). However, a decline in viable bacteria occurred 8 h post-inoculation, reaching statistical significance by 12 h (Figure 3C). Moreover, a similar decrease in bacterial viability was observed in cultures initially treated with resorufin (Figure 3C). No significant differences in viable bacteria were observed between *F. tularensis* cultures treated with either resazurin or resorufin at any of the time points (Figure 3C). Because resazurin and resorufin both exhibit a similar antimicrobial effect on *F. tularensis*, this suggests that the redox reaction itself is not responsible for the observed bactericidal activity. However, the data indicating that resazurin is rapidly converted to resorufin, while the drop in viability occurs most drastically after 8 h, may suggest that resorufin is the biologically active form responsible for decreasing the viability of *F. tularensis* LVS bacteria.

### Resazurin limits intracellular replication of *F. tularensis* in phagocytic and non-phagocytic cells

**Figure 4:**
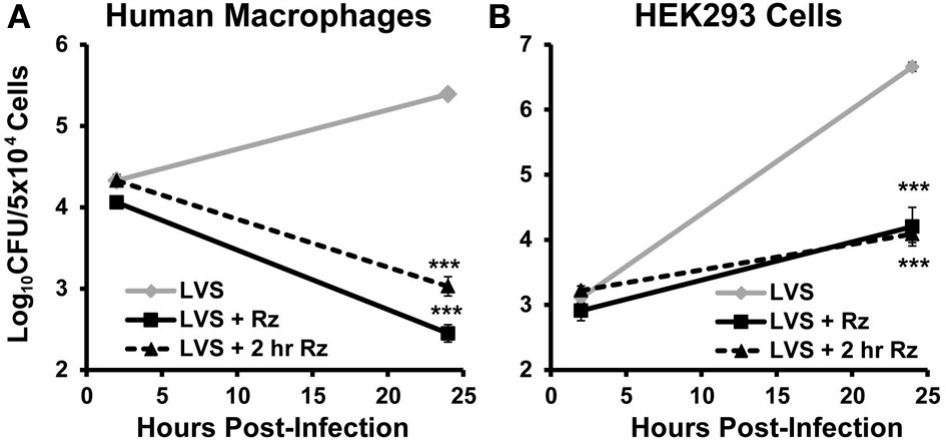
**Resazurin exhibits antimicrobial activity on intracellular *F. tularensis* LVS**. Primary human macrophages **(A)** or HEK293 **(B)** cells were infected with *F. tularensis* LVS bacteria using a gentamicin protection assay. Cells were treated with 44 μM resazurin during the entire assay (+ Rz), at 2 h post-infection (+ 2 h Rz), or were untreated. Data shown are mean ± s.e.m. and representative of three independent experiments. The limit of detection was 100 CFU. Statistically significant differences in growth at 24 h post-infection were determined by Two-Way ANOVA with Dunnett's *post-hoc* test (^***^p < 0.001).

In an infected host, *F. tularensis* resides and replicates inside macrophages (Elkins et al., 2007). Therefore, resazurin must be able to limit intracellular growth of this bacterium in these cells in order to be an effective therapeutic. To test this, primary human macrophages were infected with *F. tularensis* LVS, and viable intracellular bacteria were quantified using a gentamicin protection assay as described previously (Horzempa et al., 2008a, 2010). Cells were treated with 44 μM resazurin during the entire assay (+Rz), beginning 2 h post-infection (+2 h Rz), or left untreated. Both resazurin treatments resulted in a significant decrease in viable *F. tularensis* LVS bacteria over 22 h (Figure 4A). Visible observation of the macrophages 24 h post-infection indicated resazurin was reduced to resorufin, although this phenomenon was not quantified (data not shown). This suggested that the macrophages were still viable, and that the combination of resazurin and *F. tularensis* LVS did not culminate in undesirable toxicity. *F. tularensis* is also capable of infecting and replicating in non-phagocytic cells like epithelial cells and hepatocytes which is sufficient for pathogenesis (Horzempa et al., 2010). We next determined whether resazurin was also capable of inhibiting growth of *F. tularensis* LVS in non-phagocytic cells. To test this, a human kidney epithelial cell line HEK293 was used as a model for non-phagocytic cells. Similar to the results obtained with macrophages, treatment with resazurin significantly reduced the number of viable *F. tularensis* LVS bacteria in HEK293 cells 22 h post-infection (Figure 4B). In an additional experiment, no viable *F. tularensis* LVS bacteria were detected from HEK293 cells at 72 h post infection following treatment with 44 μM resazurin (data not shown). Based on these data, resazurin exhibits antimicrobial activity against intracellular *F. tularensis* bacteria.

## Discussion

The prevalence of antibiotic resistance in today's society highlights the need for new classes of antibiotics (Bassetti et al., 2013). Here, we identified an unanticipated bactericidal activity for a compound commonly used to measure cellular viability. Resazurin inhibited growth of only *F. tularensis* and *Neisseria* species *in vitro*, notably the human pathogens, type A *F. tularensis* (Schu S4) and *N. gonorrhoeae*. The fact that resazurin targets such a limited array of pathogenic organisms is extremely desirable from the standpoint of limiting the potential of drug resistance in the future.

**Figure 5:**
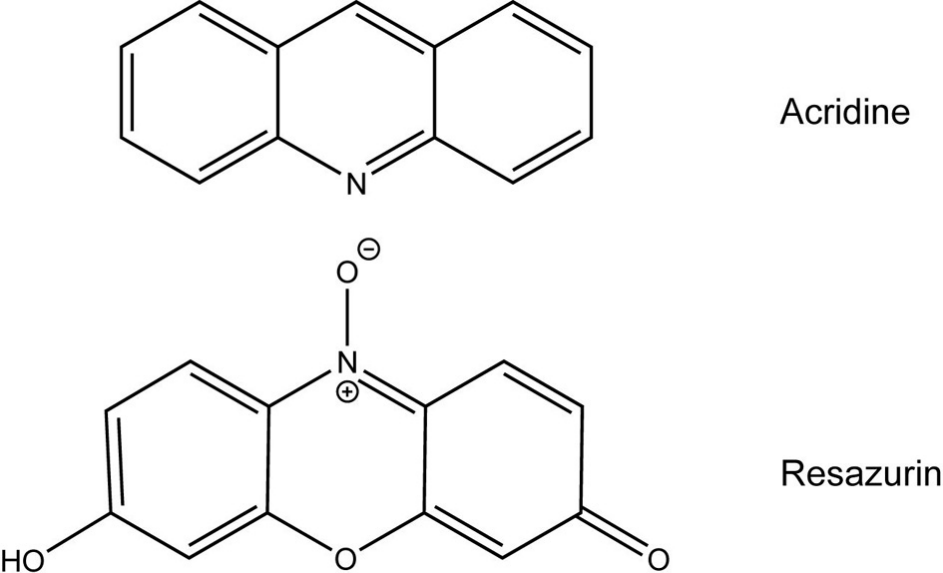
**Chemical structures of resazurin and acridine**.

Most antibiotics target pathways that are conserved by numerous bacterial species like cell wall or protein synthesis (Lewis, 2013). Resazurin is unique in that it only exhibits antimicrobial activity against two types of bacteria tested in this work, *F. tularensis* and *Neisseria* (Table 2). Aside from their fastidious nature, there are no apparent similarities between these two groups of bacteria that distinguish them from the other bacterial genera tested to suggest a mechanism of action. In culture, resazurin is reduced to resorufin by *F. tularensis* LVS, however, this chemical reaction is not responsible for the decline in viability since both compounds are equivalently bactericidal (Figure 3). Examination of the chemical structure of resazurin elucidated similarities to acridine (Figure 5). Many acridine derivatives were used as antibacterial agents during World War II (Wainwright, 2001). The planar area of the tricyclic acridine nucleus allows for intercalation of DNA resulting in its bactericidal activity (Wainwright, 2001). The possibility that resazurin functions in a similar fashion is currently being investigated.

In the human host, *F. tularensis* and *N. gonorrhoeae* reside inside host cells (Post et al., 2002; Oyston, 2008; Horzempa et al., 2011). Therefore, resazurin must be able to penetrate infected host cells to maintain its antibacterial activity *in vivo*. During the *in vitro* infection assays, resazurin was capable of limiting intracellular replication of *F. tularensis* in both phagocytic and non-phagocytic cells (Figure 4). While resazurin was converted to resorufin in these assays, the reduction of this compound by the host cells did not decrease the potency of the drug, which is consistent with the data indicating that both derivatives are bactericidal (Figures 3, 4). Resazurin has been tested for toxicity in mice at a dose 100-fold higher than that used in this study and shown to be relatively well-tolerated (Lutty, 1978). Another recently developed antibacterial compound, fosmidomycin that was effective at killing intracellular *Francisella* bacteria *in vitro*, also prolonged survival following a lethal challenge in an animal model (McKenney et al., 2012). The data presented here strongly suggest that resazurin will be an effective therapeutic for use during an *in vivo F. tularensis* or *Neisseria* infection, and substantiate the need for pre-clinical animal trials using mouse models of infection.

## Author contributions

Deanna M. Schmitt, Gerard J. Nau, and Joseph Horzempa conceived and designed the experiments. Joseph Horzempa, Deanna M. Schmitt, Dawn M. O'Dee, Brianna N. Cowan, James W.-M. Birch, and Leanne K. Mazzella performed the experiments. Deanna M. Schmitt, Joseph Horzempa, Gerard J. Nau, and Dawn M. O'Dee analyzed the data. Deanna M. Schmitt and Joseph Horzempa wrote the paper.

### Conflict of interest statement

The authors acknowledge that Joseph Horzempa, Dawn M. O'Dee, and Gerard J. Nau have submitted a patent on the antimicrobial activity of the compounds used in this manuscript. The authors declare that the research was conducted in the absence of any commercial or financial relationships that could be construed as a potential conflict of interest.
